# Image-derived and physiological markers to predict adequate adenosine-induced hyperemic response in Rubidium-82 myocardial perfusion imaging

**DOI:** 10.1007/s12350-022-02906-9

**Published:** 2022-02-11

**Authors:** Martin Lyngby Lassen, Mads Wissenberg, Christina Byrne, Majid Sheykhzade, Preetee Kapisha Hurry, Anne Vibeke Schmedes, Andreas Kjær, Philip Hasbak

**Affiliations:** 1 Department of Clinical Physiology, Nuclear Medicine & PET and Cluster for Molecular Imaging, of Biomedical Sciences, Section 4011, Rigshospitalet, University of Copenhagen, Blegdamsvej 9, 2100 Copenhagen, Denmark; 2 Department of Cardiology, Copenhagen University Hospital, Gentofte, Denmark; 3 Department of Drug Design and Pharmacology, Faculty of Health and Medical Sciences, University of Copenhagen, Copenhagen, Denmark; 4 Department of Biochemistry and Immunology, Lillebaelt Hospital, Vejle, Denmark

**Keywords:** Myocardial flow reserve, Cardiac PET, 82-Rubidium, Pharmacological stress, Adenosine

## Abstract

**Aims:**

This study aimed to investigate the potential of different markers to identify adequate stressing in subjects with and without caffeine intake prior to Rubidium-82 myocardial imaging.

**Methods and Results:**

This study comprised 40 healthy subjects who underwent four serial Rubidium-82 rest/adenosine stress MPI; two with 0mg caffeine consumption (baseline MPIs) and two with controlled consumption of caffeine (arm 1: 100 and 300mg, or arm 2: 200 and 400mg). We report the sensitivity and specificity of seven markers ability to predict adequate adenosine-induced hyperemic response: (1) the splenic response ratio (SRR); (2) splenic stress-to-rest intensity ratios (SIR); (3) changes in heart rate (ΔHR); (4) percentwise change in heart rate (Δ%HR); (5) changes in the rate pressure product (ΔRPP); (6) changes in the systolic blood pressure (ΔSBP); and (7) changes in the cardiovascular resistance (ΔCVR). Adequate stressing was determined as stress myocardial blood flow > 3ml/g/min and a corresponding myocardial flow reserve >68% of the individual maximum myocardial flow reserve obtained in the baseline MPIs.

**Results:**

129 MPI sessions (obtained in 39 subjects) were considered for this study. The following sensitivities were obtained: SSR = 72.7%, SIR = 63.6%, ΔHR = 45.5%, Δ%HR = 77.3%, ΔRPP = 54.5%, ΔSBP = 47.7%, and ΔCVR =40.9%, while the specificities were SSR = 80.9%, SIR = 85.0%, ΔHR = 90.4%, Δ%HR = 81.6%, ΔRPP=81.1%, ΔSBP = 86.4%, and ΔCVR =90.4%.

**Conclusion:**

The image-derived and physiological markers all provide acceptable sensitivities and specificities when patients follow the caffeine pausation before MPI. However, their use warrants great care when caffeine consumption cannot be ruled out.

**Supplementary Information:**

The online version contains supplementary material available at 10.1007/s12350-022-02906-9.

## Introduction

Myocardial perfusion imaging (MPI) is a common test, where Rubidium-82 (^82^Rb) has become the most used tracer in Positron Emission Tomography studies as it can be used in centers without cyclotrons.^1,2^ The accuracy of the stress myocardial blood flow and reserve assessments, however, is significantly lowered if adequate pharmacological stressing is not achieved.^3^ Previous studies have suggested that 3-20% of all stress MPI might have false-negative findings caused by inadequate pharmacological stressing of the patients.^3–5^ The most frequently used pharmacological stressors are regadenoson, dipyridamole, and adenosine; they all activate the adenosine receptor A_2A_ in coronary arteries and initiate a dilatation of these vessels, which in turn increases myocardial blood flow up to fourfold.^6^ Coffee, caffeine, and other methylxanthines act as non-selective antagonists at the adenosine receptors and are expected to dose-dependently attenuate vasodilator-induced myocardial hyperaemia and may, therefore, reduce the sensitivity of radionuclide MPI for the detection of inducible perfusion abnormality in patients with coronary artery disease.^7^ The easy identification of adequate hemodynamic response in the patients persists to be a problem; however, several physiological, image-derived, and hybrid markers have been suggested to identify the adequate response.^3,8,9^ This study aimed to evaluate seven of the proposed markers ability to predict adequate adenosine-induced hyperemic response in subjects with or without caffeine consumption prior to MPI. The seven markers can be classified as image-derived markers (splenic response ratio (SRR) and splenic stress-to-rest intensity ratios (SIR)) and markers of changes in physiological response between rest and stress MPI (heart rate (ΔHR), the percentwise change in HR (Δ%HR), changes in the rate pressure product (ΔRPP), and changes in the systolic blood pressure (ΔSBP)). Finally, we evaluate a hybrid marker employing both image data and physiological measures (changes in the cardiovascular resistance between rest and stress MPI (ΔCVR)).

## Materials and Methods

### Study Population

This study comprised 40 young, healthy subjects (median age = 23, interquartile range = [22, 25], 19 females) recruited for ^82^Rb rest/stress MPI in a hybrid PET/CT system. Each subject had four serial MPI sessions within 27 days (interquartile range = 17-36 days), acquired with and without controlled caffeine consumption before the MPI session. Inclusion criteria were age > 18 years, no participation in studies testing drugs, no regular consumption of medicine, no known medical condition, and no use of tobacco and euphoric substances (except alcohol) within three months prior to study participation. Exclusion criteria were pregnancy, allergy, intolerance to theophylline or adenosine, any prior medical history of asthma, and inability to adhere to the study protocol. The Scientific Ethics Committee of the Capital Region of Denmark [protocol number H-15009293] and the Danish Data Protection Agency approved this study, and all volunteers provided informed oral and written consent.

### Imaging Protocol

#### Positron emission tomography acquisition

**Figure 1:**
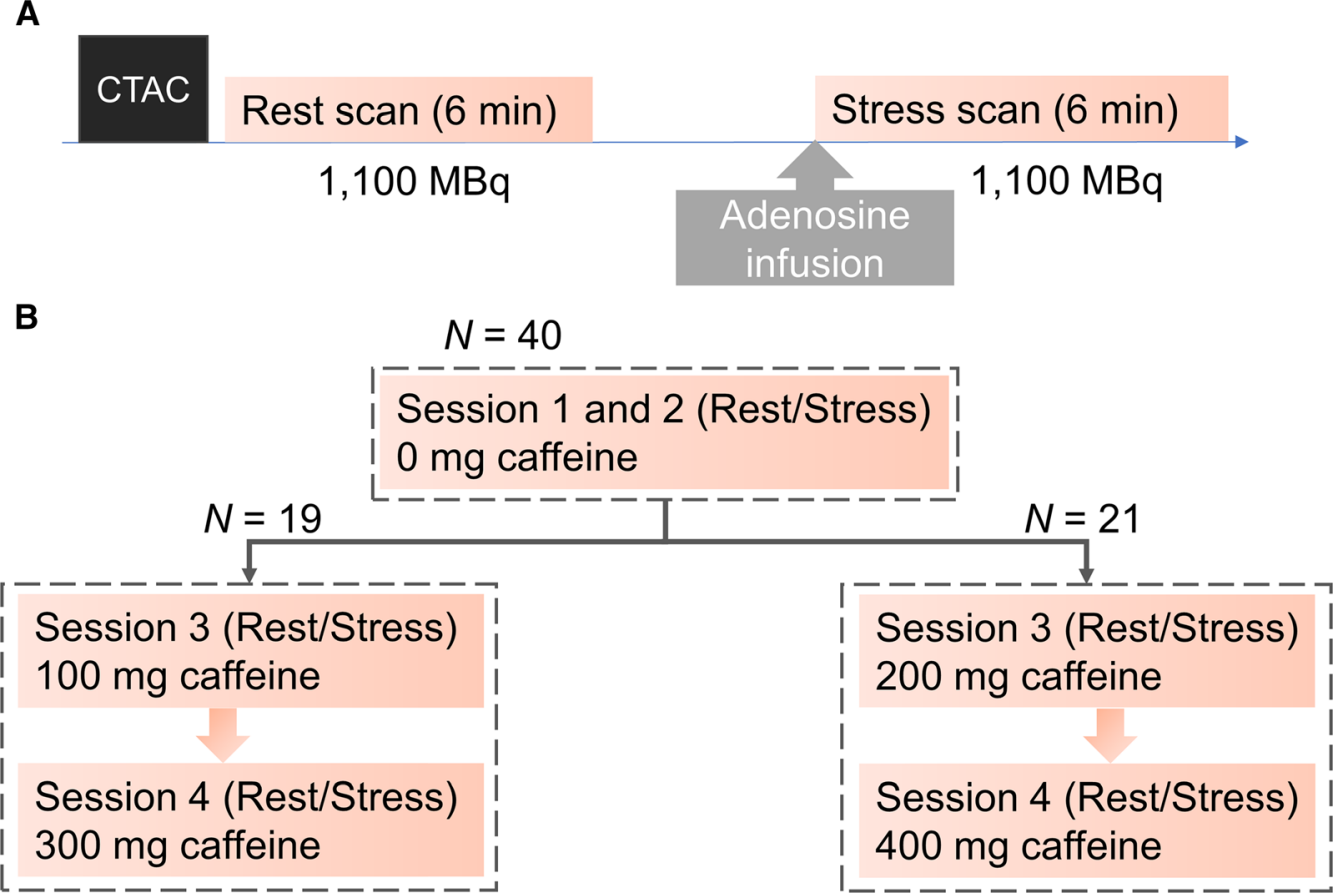
**a** MPI protocol for each of the 4 PET/CT MPI sessions. **b** Study protocol for the 40 healthy subjects. *PET* positron emission tomography, *CT* computed tomography, *CTAC* CT attenuation correction

The subjects were divided into two groups, both groups having four serial ^82^Rb MPI sessions, each consisting of an ^82^Rb rest/stress imaging protocol (Figure 1). For each acquisition, the subjects had a target injection dose of 1,100MBq (30mCi) ^82^Rb in a 128 slice positron emission tomography/computed tomography system (Siemens Biograph mCT). Pharmacological stressing was obtained using adenosine infused at 140 mg/kg/min for six minutes, with the emission acquisition starting 2.5 minutes into the infusion. The subjects were instructed to abstain from caffeine at least 24 hours before each of the four MPI sessions to comply with the controlled caffeine consumption setup. Each study subject had two MPI sessions obtained without controlled caffeine intake before the MPI sessions (baseline MPIs) and two with controlled caffeine intake. For the two MPI sessions with controlled caffeine intake, one half had an oral intake of 100mg and 300 caffeine (arm 1), while the other half had an oral intake of 200mg and 400mg caffeine (Figure 1). For the controlled caffeine intake MPI’s, the caffeine was provided as tablets diluted into hot water, taken orally 1hr before the rest acquisition. Plasma caffeine concentrations (PCC) were evaluated at the time of the stress MPI by averaging measurements obtained at 75 and 90 min post ingestion of caffeine, using high-performance liquid chromatography–mass spectrometry (LC-MS/MS).

#### Positron emission tomography reconstruction protocol and data processing

Four image series were employed in the study; two dynamic image series used for assessments of myocardial blood flow and reserve, and two static image series used for ratio analyses (SRR, SIR, and ΔCVR). The dynamic image series were reconstructed into 18 frames (1 x 10s, 8 x 5s, 3 x 10s, 2 x 20s, 4 x 60s) using an iterative 3D ordered subset expectation maximization model with corrections for time-of-flight and point-spread function employing 2iterations, 21subsets. The static images were reconstructed utilizing data from 3.5 minutes, using an injection-to-scan delay of 2.5min. Both image series had a 6.5mm Gaussian post filtering of the data. Myocardial blood flow was calculated using the Lortie model,^10^ while the myocardial flow reserve (MFR) was calculated as the ratio between the rest and stress blood flows. Both myocardial blood flow and MFR values were obtained in dedicated software (QPET, Cedars-Sinai Medical Center, Los Angeles, California, USA).

#### Image-derived markers

**SRR** was calculated using a previously described method, employing static rest and stress ^82^Rb perfusion images^11^ (Eq. 1).

1 $$SSR=\frac{{Stress}_{Spleen} / {Stress}_{Liver}}{{Rest}_{Spleen} / {Rest}_{Liver}}$$

**Figure 2:**
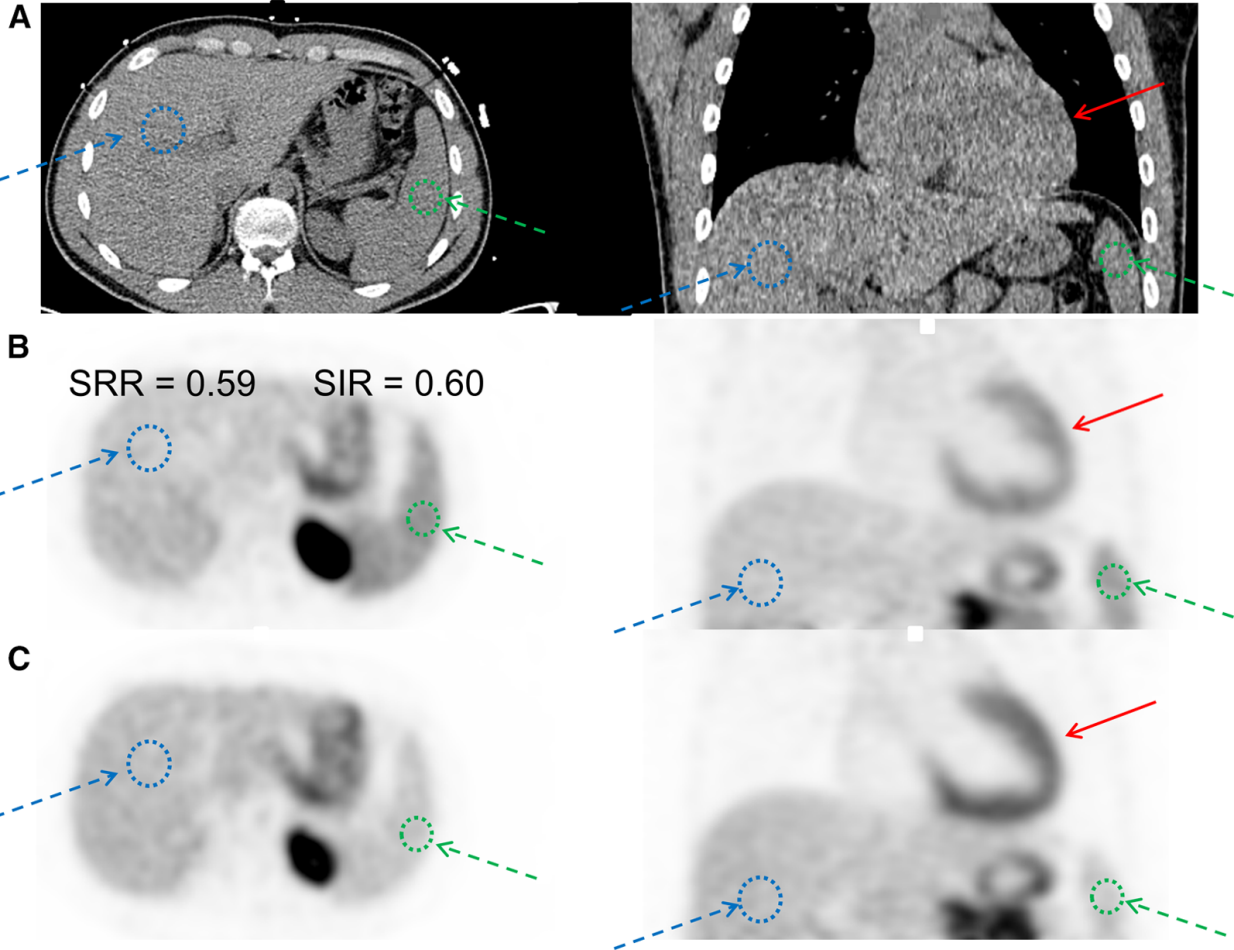
Identification of SRR and SIR. Two volumes of interest were in the spleen (sphere, *r* = 20 mm − green arrow) and liver (sphere, *r* = 50 mm − blue arrow). Anatomical identification of the spleen and heart (red arrow) was obtained using the low-dose CT images acquired for attenuation correction purposes (**a**). In **b** and **c** we show the rest and stress uptake parameters for a scan with concordant assessment of adequate hyperemic response as obtained using both SRR and SIR. *SRR* splenic response ratio, *SIR* splenic stress-to-rest intensity ratio

Rest_Spleen_ and Stress_Spleen_ represent the mean standardized uptake values obtained in the spleen at rest and stress using a 20 mm spherical volume of interest (VOI), respectively; Rest_Liver_ and Stress_Liver_ represent the mean rest and stress standardized uptake values obtained in the liver using a 50 mm spherical VOI (Figure 2).^12^ The normal limits of SRR were calculated using a previously described method, using the mean + 1 standard deviation (SD).^12^ Only baseline MPIs with PCC <1.0mg/l were considered to ensure the normal range was representative for the guideline-recommended withdrawal of caffeine before stress MPI sessions. In this study, SRR ≤ 0.75 were considered to have splenic activation (group average SRR = 0.59, SD = 0.16).

Using the same segmentations, SIR was calculated by normalizing the stress ^82^Rb uptake observed in the spleen to the activity observed during rest (Eq. 2):

2 $$SIR=\frac{{Stress}_{Spleen} }{{Rest}_{Spleen}}$$

The cut-off value was obtained using an average SIR obtained for the same baseline MPIs + 1SD as used for the SRR calculations. SIR ≤ 0.83 were considered to have adequate stressing (group average SIR = 0.70, SD = 0.13).

#### Physiological markers

The cut-off values obtained for the physiological markers all rely on measurements obtained for the baseline scans, with PCC < 1.0mg/L.

Peak HR for rest and stress scans using the ECG trigger signals were obtained by averaging the three fastest consecutive beats obtained using the three lead ECG-triggering system. Increases in the peak heart rate (ΔHR) ≥ 18 min^-1^ were considered a marker for adequate stressing of the subjects, calculated as the average increase in HR – 1SD (average increase in HR = 29, SD= 11). In addition, a percentwise increase in the HR from rest to stress was reported (Δ%HR) using a cut-off value of 25%, calculated as the average percentwise increase − 1SD (average change = 43%, SD = 18%).

Rate pressure products (RPP) were calculated for all MPI sessions defined as RPP = systolic blood pressure)*(Heart rate). Increases in the RPP ≥1,250 mmHg * min^-1^ from rest to stress MPI was considered a marker for adequate stress using a cut-off value determined as the mean change in RPP – 1SD; ΔRPP ≥ 1250 (average = 3012, SD=1733).

Changes in the systolic blood pressure (ΔSBP) were calculated for all MPI sessions defined as the systolic rest blood pressure – systolic stress blood pressure. Decreases in the systolic blood pressure ≥38mmHg from rest to stress MPI was considered a marker for adequate stress using a cut-off value determined as the mean change in the systolic pressure – 1SD; ΔSBP ≥ 38 (average = 48, SD = 10).

#### Hybrid markers

Cardiovascular resistance was obtained for all MPIs using Eq. 3.

3 $$CVR=0.33\times \frac{(2\times\;diastolic\;blood\;pressure +systolic \;blood\;pressure)}{Myocardial\;blood\;flow}$$

For the rest MPI sessions, diastolic and systolic blood pressures were acquired immediately before the emission acquisition, while they were measured 2-2.5 min into the adenosine infusion and, thus, immediately before ^82^Rb infusion start for the stress MPI sessions. The cut-off for normal changes in the cardiovascular resistance (ΔCVR) was considered as mean ΔCVR – 1SD obtained for baseline MPIs with repeat PCC <1.0mg, introducing a cut-off on 40% reduction (mean = 68%, SD = 28). The unit for the cardiovascular resistance is mmHg/mL/g/min.

#### MFR: normal range and reproducibility

The healthy subjects were expected to have normal MFR (≥ 2.5); thus, the usual thresholds for normal flow reserve could not be used.^13^ To ameliorate the anticipated increased MFR values, we introduced a population-based cut-off level based on the hyperemic responses obtained in patients with both repeat scans PCC < 1.0 mg. For these patients, we normalized the repeated MFR values to the intra-subject highest MFR. Using only interscan variation values, we obtained the normal interscan variation in MFR defined as the average interscan difference for the baseline MPIs – 1SD (average off-set = 83.0%, SD = 15.0%; cut-off = 68%). In this context, sufficient hyperemic response was only determined with stress myocardial blood flow > 3ml/g/min and MFR ≥ 68% of the individual maximum obtained MFR during the baseline scans.

*Sensitivity, specificity, and positive and negative predictive values* were calculated for all seven markers using confusion matrices. In brief, the sensitivity is calculated as (true positive measures)/((true positive measures) + (false-negative measures)), specificity is calculated as (true negative measures)/((true negative measures)+(false positive measures)). Positive predictive values are calculated as (true positive measures)/((true positive measures)+(false positive measures)), and negative predictive value as (true negative measures)/ ((true negative measures )+(false-negative measures)).

### Statistical Analysis

Data were quantified in R (The GNU project). For descriptive analyses, we used mean ± standard deviation, range or median, and interquartile range for continuous values. Differences for the markers and their dependencies on PCC were calculated using multivariate ANOVA analyses. Two-tailed *p* values less than 0.05 were considered statistically significant. All data were checked for normality using the Shapiro-Wilk test.

## Results

Of the forty healthy subjects included in this study, 24 MPI sessions were excluded because of; non-compliance with the study protocol (*N* = 4, 1 subject), breath-hold during the low-dose computed tomography scans acquired for attenuation correction purposes (shifting of the myocardium on PET and CT with consequent changes in the anatomical position of diaphragm and lungs, rendering it impossible to obtain acceptable co-registration of the datasets) (*N* = 5), significant motion during the emission scans (*N* = 4), incomplete physiological measures (*N* = 4), and missing emission raw data (listmode data) (*N* = 7). Finally, 7 of the baseline MPI sessions (0mg caffeine ingested) were reported to have PCC ≥ 1.0 mg/L (PCC = 4.3 ± 3.3 mg/L) and, thus, not considered in this study. In total, 129 of the acquired 160 MPI sessions were deemed acceptable for further studies (80.6% of the MPI sessions) (0mg MPI: 63, 100 mg: 19, 200 mg: 15, 300 mg: 16, 400 mg: 16). PCC obtained for the five caffeine ingestion protocols is shown in Table 1.

**Table 1:** PCC obtained for MPI sessions with and without consumption of caffeine. N indicates the number of MPI sessions acquired using the respective caffeine doses. Significant differences in the PPC were reported for the two genders for all 5 caffeine consumption groups.

PCC (mg/l)					
	0mg caffeine given (*N* = 63)	100mg caffeine given (*N* = 19)	200mg caffeine given (*N* = 15)	300mg caffeine given (*N* = 16)	400mg caffeine given (*N* = 16)
Females	0.3 ± 0.2 (*N* = 28)	2.7 ± 0.6 (*N* = 9)	6.0 ± 2.4 (*N *= 7)	8.2 ± 0.1 (*N *= 8)	13.1 ± 5.5 (*N *= 9)
Males	0.1 ± 0.2 (*N* = 35)	1.4 ± 0.3 (*N* = 10)	5.2 ± 0.6 (*N *= 8)	4.6 ± 0.9 (*N *= 8)	7.0 ± 1.3 (*N *= 7)
Grouped	0.2 ± 0.2	2.0 ± 0.7	5.5 ± 2.4	6.4 ± 2.1	10.4 ± 5.2

*PCC* plasma caffeine concentration

**Figure 3:**
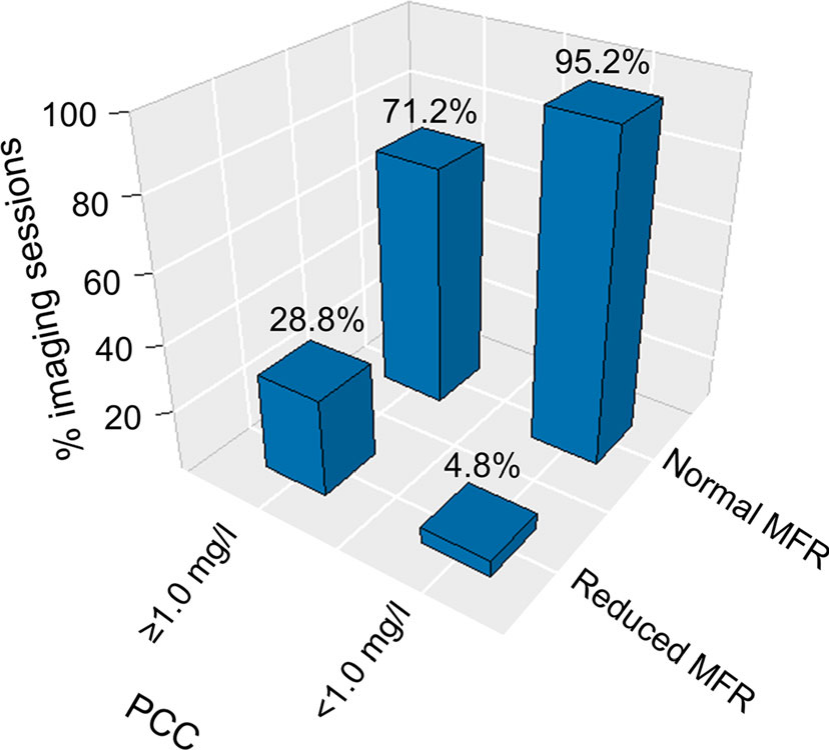
Bar plot of normal vs. reduced MFR according to caffeine concentration. Normal MFR was declared when the MFR was ≥ 68% of the maximum MFR obtained for the baseline MPIs. *MFR* myocardial flow reserve, *PCC* plasma caffeine concentration

Employing the cohort-specific threshold for normal MFR, adequate hyperemic response was obtained in 95.2% of the 63 accepted MPI sessions with caffeine concentrations < 1.0 mg/l; and 71.2% of the 66 accepted MPI sessions with caffeine concentrations ≥1.0mg/l (Figure 3). In supplementary material 1, we show the normal ranges and the respective cut-off values obtained for the baseline scans (with repeat PCC < 1.0 mg/L) for all seven markers and the normalized MFR values.

*MPI sessions* with caffeine concentrations < 1.0 mg/l

**Figure 4:**
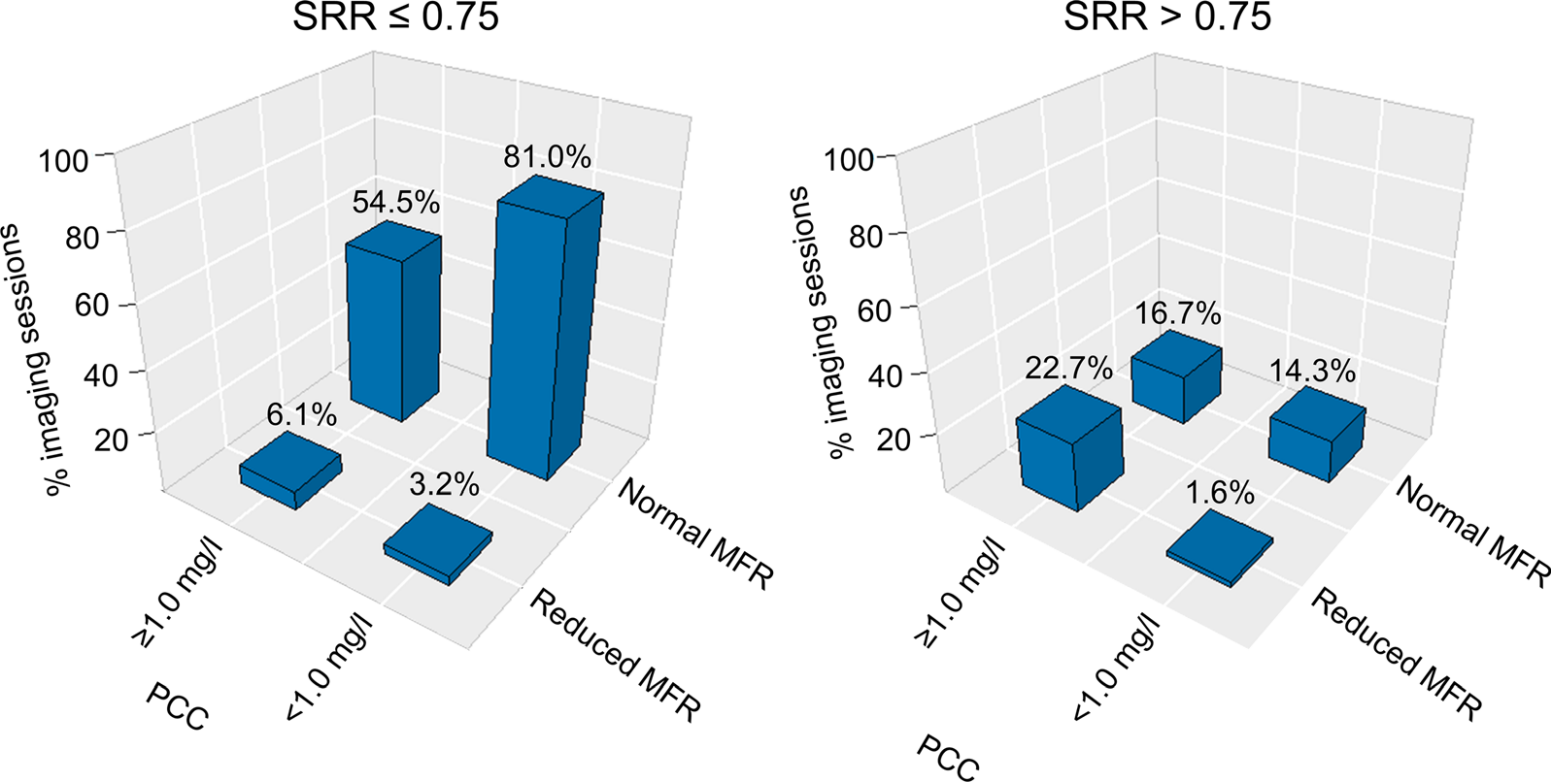
Bar plot of studies with and without adequate hemodynamic response when employing SSR, stratified by PPC. Adequate hemodynamic response was declared for SRR ≤ 0.75. The numbers above bars indicate the percentwise number of occurrences for the respective measures. *SRR* splenic response ratio, *MFR* myocardial flow reserve, *PCC* plasma caffeine concentration

**Figure 5:**
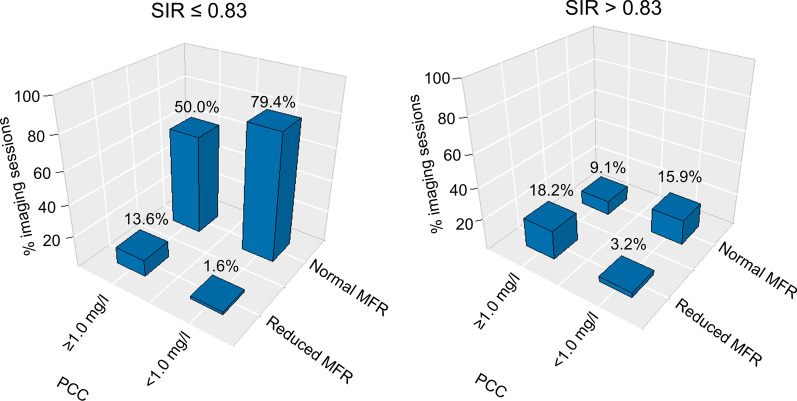
Bar plot of studies with and without consideration of adequate hemodynamic response when employing SIR. Adequate hemodynamic response was declared for SIR ≤ 0.83. The numbers above bars indicate the percentwise number of occurrences for the respective measures. The data were divided into two subgroups, studies with and without significant plasma concentrations of caffeine (PCC ≥ 1.0 mg/l and PCC < 1.0 mg/l), respectively. *SIR* splenic stress-to-rest intensity ratio, *MFR* myocardial flow reserve, *PCC* plasma caffeine concentration, *MPI* myocardial perfusion imaging

**Figure 6:**
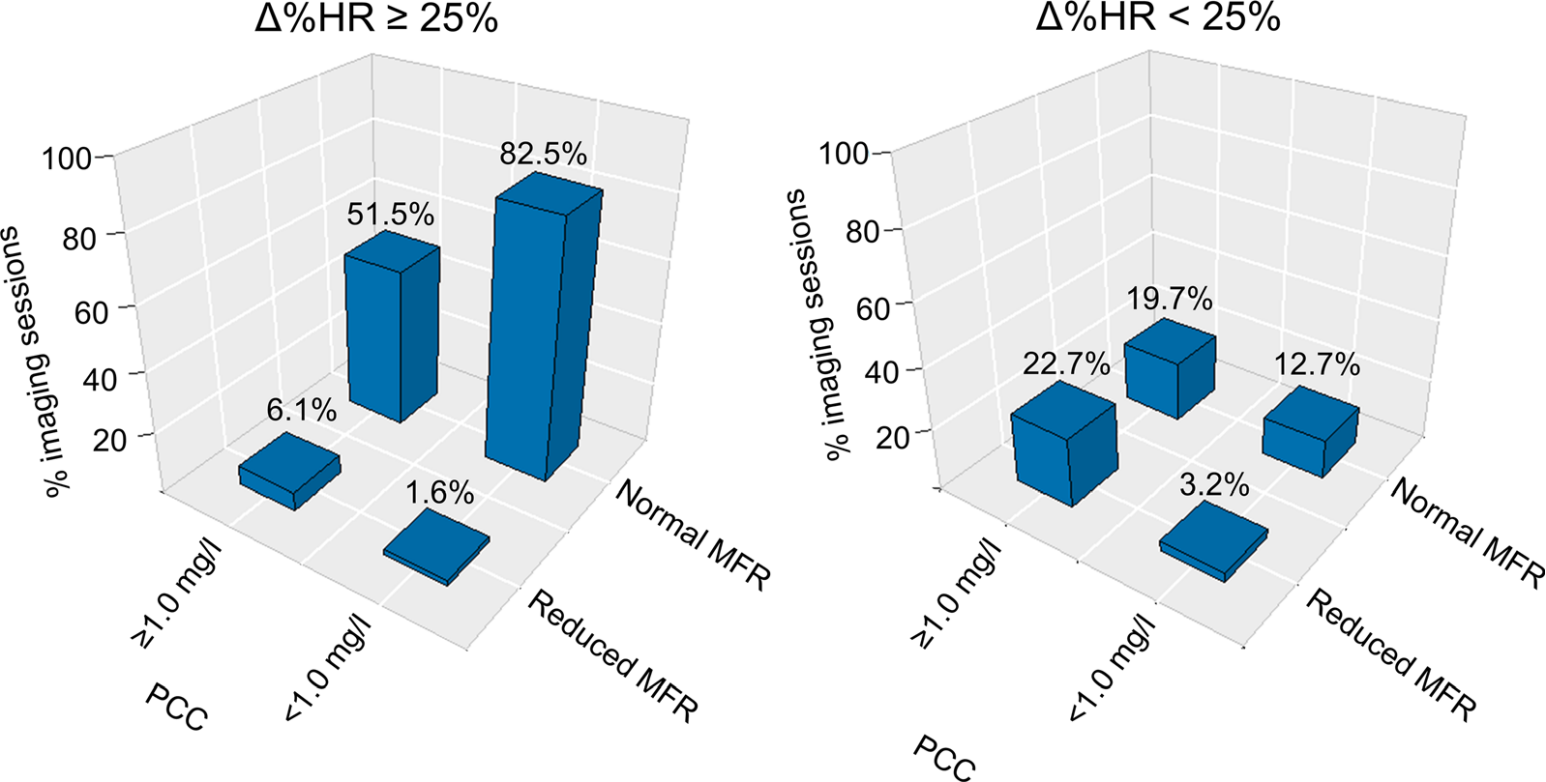
Bar plot of studies with and without consideration of having adequate hemodynamic response for Δ%HR, stratified by PPC. Adequate hemodynamic response was declared for Δ%HR≥25. The numbers above bars indicate the percentwise number of occurrences for the respective measures. *Δ%HR* percent wise change in peak heart rate as observed for the ECG trigger signals between rest and stress MPI, *MFR* myocardial flow reserve, *PCC* plasma caffeine concentration, *MPI* myocardial perfusion imaging

The average PCC was 0.2 ± 0.2 mg/l (38 subjects with 63 accepted MPIs) (Table 2). Adequate hyperemic response was observed in 95.2% (60 out of 63 MPI sessions) when employing the MFR cut-off value (MFR ≥ 68% of the maximal MFR obtained during the baseline MPI sessions), while adequate hyperemic response was predicted in 81.0% to 90.5% of the sessions (range: 51-57 out of 63 MPI sessions) when employing the seven selected markers cut-off values (Table 3). Bar plots of normal versus reduced MFR according to the cut-off value of SSR, SIR, and Δ%HR, and stratified to caffeine concentration, are shown in figures 4-6. Similar barplots for ΔHR, ΔRPP, ΔSBP, and ΔCVR are shown in supplementary material.

**Table 2:** MPI measurements and performance of the seven markers used for testing of adequate hyperemic response. Significant differences were observed for four of seven markers when the PCC was increased to ≥ 1.0mg/L. changing, marked in bold (all *p* < 0.05).

	<1.0 mg/l	≥ 1.0 mg/l
Number of subjects (MPI sessions)	38 (63)	39 (66)
MFR	3.80 ± 0.87	**3.39 ± 1.21 (*p* = 0.027)**
PCC (mg/l)	0.2 ± 0.2	**5.9 ± 4.2 (*p* < 0.001)**
Stress markers		
SRR	0.59 ± 0.15	**0.70 ± 0.16 (*p* < 0.001)**
SIR	0.70 ± 0.13	0.75 ± 0.14
ΔHR	29 ± 13	**46 ± 38 (*p* < 0.001)**
Δ%HR	44 ± 21	**23 ± 37 (*p* = 0.001)**
ΔRPP	3,091 ± 1,730	**1,075 ± 5080 (*p* = 0.003)**
ΔSBP	49 ± 12	**2 ± 14 (*p* < 0.001)**
ΔCVR	69 ± 26	62 ± 32

*PCC* plasma caffeine concentration, *SRR* splenic response ratio, *SIR* splenic stress-to-rest intensity ratio, *ΔHR and Δ%HR* the change in measured and the percentwise heart rate from rest to stress MPI, respectively. *ΔRPP* the change in the rate pressure product from rest to stress MPI. *ΔSBP* change in the systolic blood pressure from rest to stress, *ΔCVR* the change in the cardiovascular resistance from rest to stress MPI. *MFR* myocardial flow reserve

**Table 3:** Number of subjects considered to have adequate pharmacological stressing as a function of the PCC. Numbers in parenthesis indicate percentwise population.

	SRR	SIR	ΔHR	Δ%HR	ΔRPP	ΔSBP	ΔCVR	MFR
< 1mg/l (*N*=63)	53 (84.1%)	51 (81.0%)	53 (84.1%)	53 (84.1%)	54 (85.7%)	52 (82.5%)	57 (90.5%)	60 (95.2%)
≥ 1mg/l (*N*=66)	40 (60.6%)	48 (72.7%)	56 (84.8%)	38 (57.6%)	45 (68.2%)	2 (3.0%)	54 (81.8%)	47 (71.2%)

*PCC* plasma caffeine concentration, *SRR* splenic response ratio, *SIR* splenic stress-to-rest intensity ratio, *HR* heart rate, *RPP* rate pressure product, *SBP* systolic blood pressure, *CVR* cardiovascular resistance, *MFR* myocardial flow reserve, *Δ* changes between rest and stress MPI

*MPI sessions* with caffeine concentrations ≥ 1.0mg/l

The average PCC were 5.9 ± 4.2 mg/l (36 subjects with 66 accepted MPIs) (Table 2). Adequate hyperemic response was observed in 71.2% (47 of 66 MPI sessions) when employing the MFR cut-off value (MFR ≥ 68% of the maximal MFR obtained during the baseline MPI sessions), while adequate hyperemic response was predicted in 3% to 84.8% (range: 2-56 out of 66 MPI sessions) when employing the seven selected markers cut-off values (Table 3). Bar plots of normal versus reduced MFR according to cut-off value of SSR, SIR, and Δ%HR, and stratified to caffeine concentration, are shown in figures 4-6. Similar Bar plots for ΔHR, ΔRPP, and ΔCVR are shown in supplementary material 2-4.

### Marker Sensitivities and Specificities

**Figure 7:**
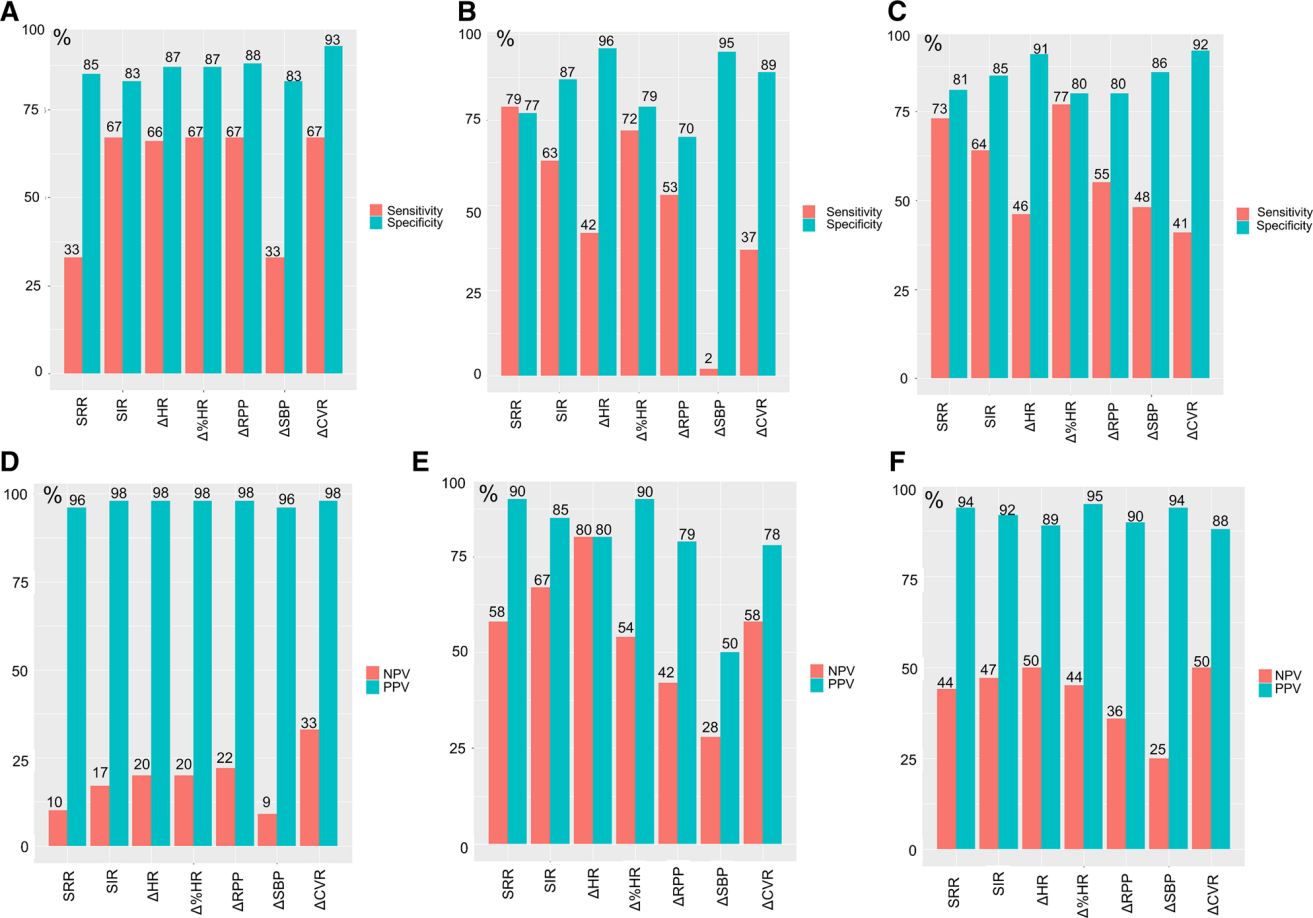
Performance measures for the seven markers used for identification of adequate stressing of the volunteers. Sensitivity and specificity for the data obtained with PCC<1.0 and PCC≥1.0mg/l are shown in **a** and **b** respectively. The grouped measures (PCC<1.0 and PCC≥1.0mg/l) are shown in **c** PPV and NPV are shown for the datasets with PCC<1.0mg/L and PCC≥1.0mg/l in **d** and **e** respectively. In **f** the NPV and PPV are shown for the grouped measures (PCC<1.0 and PCC≥1.0mg/l). *SRR* splenic response ratio, *SIR* splenic stress-to-rest intensity ratio, *ΔHR* change in heart rate between rest and stress MPI, *Δ%HR* percentwise change in heart rate between rest and stress MPI, *ΔRPP* change in rate pressure product, *ΔSBP* change in systolic blood pressure, *ΔCVR* change in cardiovascular resistance between rest and stress MPI. *NPP* negative predictive value, *PPV* positive predictive value

The sensitivity and specificity for all seven markers are shown in Figure 7a, while Figure 7b shows the positive and negative predictive values of the markers. Similar sensitivities and specificities were observed for all markers except SRR, which had reduced sensitivity, when the subjects did not have any caffeine intake prior to the MPI, while excellent PPV values were obtained for all markers (Figure 7). However, following caffeine ingestion, the sensitivities dropped for 4 of 7 markers, while SRR had a marginal gain in the sensitivity following caffeine ingestion. The caffeine was observed to reduce the positive predictive value for all seven markers, while the negative predictive values were increased.

## Discussion

This study evaluated the sensitivity and specificity of seven markers suggested to monitor adequate pharmacological stressing of patients when employing adenosine in ^82^Rb MPI sessions. In this controlled, randomized study of healthy subjects, sensitivities and specificities were acceptable for all physiological and image-derived markers. The main message from this study is that all the markers for adequate stressing should only be used with care in the clinical routine and only when the patients have followed the guideline recommendations on abstaining from caffeine for a minimum of 12 hours before the stress MPI sessions.^14^

Adequate stressing is of utmost importance to ensure reliable assessments of stress myocardial blood flow and thereby MFR. In this study, the sensitivity and specificity of seven markers were evaluated in a cohort of healthy subjects who underwent four serial MPI sessions with and without caffeine consumption prior to stress MPI (Figure 1). Caffeine (1,3,7 trimethylxanthine) is thought to competitively inhibit the adenosine receptor and thus may also attenuate the hyperemic cardiac response following a dose-dependent affinity with gender-specific responses.^7,9,15^ Reduced cardiac hyperemia is expected for patients when plasma caffeine concentrations ≥1.0mg/l when adenosine, regadenosone or dipyridamole are used as pharmaceutical stressing agents owing to caffeine's non-specific binding to the A_2A_ receptor.^4,7–9,14,16–18^ In this context, the PCC level affects the hyperemic response more strongly in men than females.^7^ In this study, reduced hyperemic responses were observed in 22 (16.2%) of the 129 accepted MPI sessions, of which 19 (86.4%) had elevated PPC (Table 3). For SRR, adequate stressing was called when the ratio was ≤0.75, obtained as the activity observed in the spleen normalized to the activity observed in the liver. This phenomenon occurs as adenosine receptor A_1_ stimulation reduces splanchnic artery perfusion resulting in splenic blood flow attenuation. For this approach to be successful, it demands that adenosine and caffeine have identical pharmacological potency at the A_1_ and A_2A_ receptor sites. This was not the case in our study, with a modest correlation between cardiac hyperemia (A_2A_) and reduced splenic perfusion (A_1_). Similarly, the same reservation applies to the SIR estimate.

The ability of adenosine to induce tachycardia is ascribed to peripheral vasodilatation, with the assumption that HR change is a direct reflection of peripheral vasodilatation. In addition, both direct and reflex baroreceptor-mediated sympathetic activation is thought to play a role.^19,20^ In a previous study it was found that a change in HR>15 beats per minute was associated with adequate stressing of the patients – a threshold lower than reported in this study (ΔHR≥18).^21^ The discrepancy in the ΔHR reported in this study when compared to Kotecha et al’s results (^21^) might be associated with the differences in the age of the study cohorts evaluated and reflects that ΔHR alone might not be fully representative for adequate stressing of the patients. However, the percentwise change in the HR provided the better sensitivity and specificity, while they had predictive values compared to ΔHR. In this study, an increase of ≥25% of the rest HR was a positive predictor that the subjects had adequate stressing during the scans. Likewise, changes in the RPP were evaluated as a marker for adequate stressing of the subjects. An increase in the RPP≥1250 was found to be an acceptable predictor of adequate stressing during the MPI sessions. Adenosine results in a modest increase in heart rate and a modest decrease in both systolic and diastolic BPs.^22^ Reductions in the SBP from rest to stress were evaluated as a marker for adequate stressing of the patients. It was found that it had an excellent sensitivity to elevated PCC values; however, it had a poor specificity to detect sufficient hyperemic response in the volunteers when ΔSBP>38 was used as a cut-off value. Finally, ΔCVR was found to offer the poorest sensitivity and specificity when caffeine consumption could not be ruled out. In this study, adequate stressing of the patients was declared when ΔCVR ≥40% in the rest scan as compared to the stress scan.

In this study, both SRR and SIR measures were in concordance with previous findings.^21,23^ Although all evaluated markers serve as acceptable indicators for adequate stress, the cut-off values are found to vary across studies.^12,21,23^ The varying cut-off values indicate that the thresholds employed are cohort-specific and, thus, a generalization of the methods is difficult. Moreover, the non-consistent cut-off values are a general limitation of all the suggested techniques, which also applies to this study. Another limitation to this study is the small cohort evaluated, consisting of 40 volunteers. Linked to the small cohort is that the volunteers are young, healthy individuals and thus not the typical patient undergoing MPI, with an intermediate likelihood of ischemic heart, which might affect the sensitivity and specificity of the markers. Finally, any cox-hazard models could not be calculated for the respective methods in this study, and, thus, the diagnostic impact of the methods cannot be determined.

## New knowledge gained

In this study, seven markers suggested for the detection of adequate hyperemic cardiac response was tested in a controlled setting, with cross-validation of the respective models. Common for all the markers is that they only provide acceptable sensitivities and specificities in a cohort of young volunteers. Therefore, their use warrants care in the clinical setting.

## Conclusion

In a cohort of young, healthy individuals, all image-derived and physiological markers provided acceptable sensitivities and specificities when the subjects followed the guideline-recommended caffeine pausation before MPI. Nevertheless, their use warrants great care when caffeine consumption prior to MPI cannot be ruled out.

## Supplementary Information

Below is the link to the electronic supplementary material.

Supplementary file1 (DOCX 1111 kb)

Supplementary file2 (PPTX 563 kb)

Supplementary file3 (TIF 8083 kb)

## Abbreviations

ΔCVR: Changes in the cardiovascular resistance between rest and stress MPI
ΔHR: Changes in heart rate between rest and stress MPI
MFR: Myocardial flow reserve
MPI: Myocardial perfusion imaging
PCC: Plasma caffeine concentration
^82^Rb: 82-Rubidium
ΔRPP: Changes in the rate pressure product between rest and stress MPI
SIR: Splenic stress-to-rest intensity ratio
SRR: Splenic response ratio
