# Supplementary material for: Image-derived and physiological markers to predict adequate adenosine-induced hyperemic response in Rubidium-82 myocardial perfusion imaging
Source: J Nucl Cardiol. 2022 Feb 11;29(6):3207–17. doi: 10.1007/s12350-022-02906-9 (PMC9834126; doi:10.1007/s12350-022-02906-9)
Supplement: Supplementary file 1 — Supplementary file1 (DOCX 1111 kb) [file 12350_2022_2906_MOESM1_ESM.docx]

**Image-derived and physiological markers to predict adequate adenosine-induced hyperemic response in Rubidium-82 myocardial perfusion imaging**

Martin Lyngby Lassen, PhD^1^, Mads Wissenberg MD^2^, PhD, Christina Byrne, MD, PhD^1^, Majid Sheykhzade, PhD^3^, Preetee Kapisha Hurry, MD^1^, Anne Vibeke Schmedes^4^, Andreas Kjær, MD, PhD, DMSc^1^, Philip Hasbak, MD, DMSc^1^

^1^Department of Clinical Physiology, Nuclear Medicine & PET and Cluster for Molecular Imaging, Department of Biomedical Sciences, Rigshospitalet and University of Copenhagen

^2^Department of Cardiology, Copenhagen University Hospital, Gentofte, Denmark.

^3^Department of Drug Design and Pharmacology, Faculty of Health and Medical Sciences, University of Copenhagen, Copenhagen, Denmark

^4^Department of Biochemistry and Immunology, Lillebaelt Hospital, Vejle, Denmark

Short title: Markers may not predict adequate adenosine stress

Corresponding Author:

Martin Lyngby Lassen, Department of Clinical Physiology, Nuclear Medicine and PET and Cluster for Molecular Imaging, section 4011, Rigshospitalet, University of Copenhagen, Blegdamsvej 9, 2100 Copenhagen, Denmark

**Supplementary material 1. Normalized MFR and marker values obtained for the repeat baseline scans with PCC<1.0mg/L.** The respective values are shown in table 2 of the main manuscript.

**
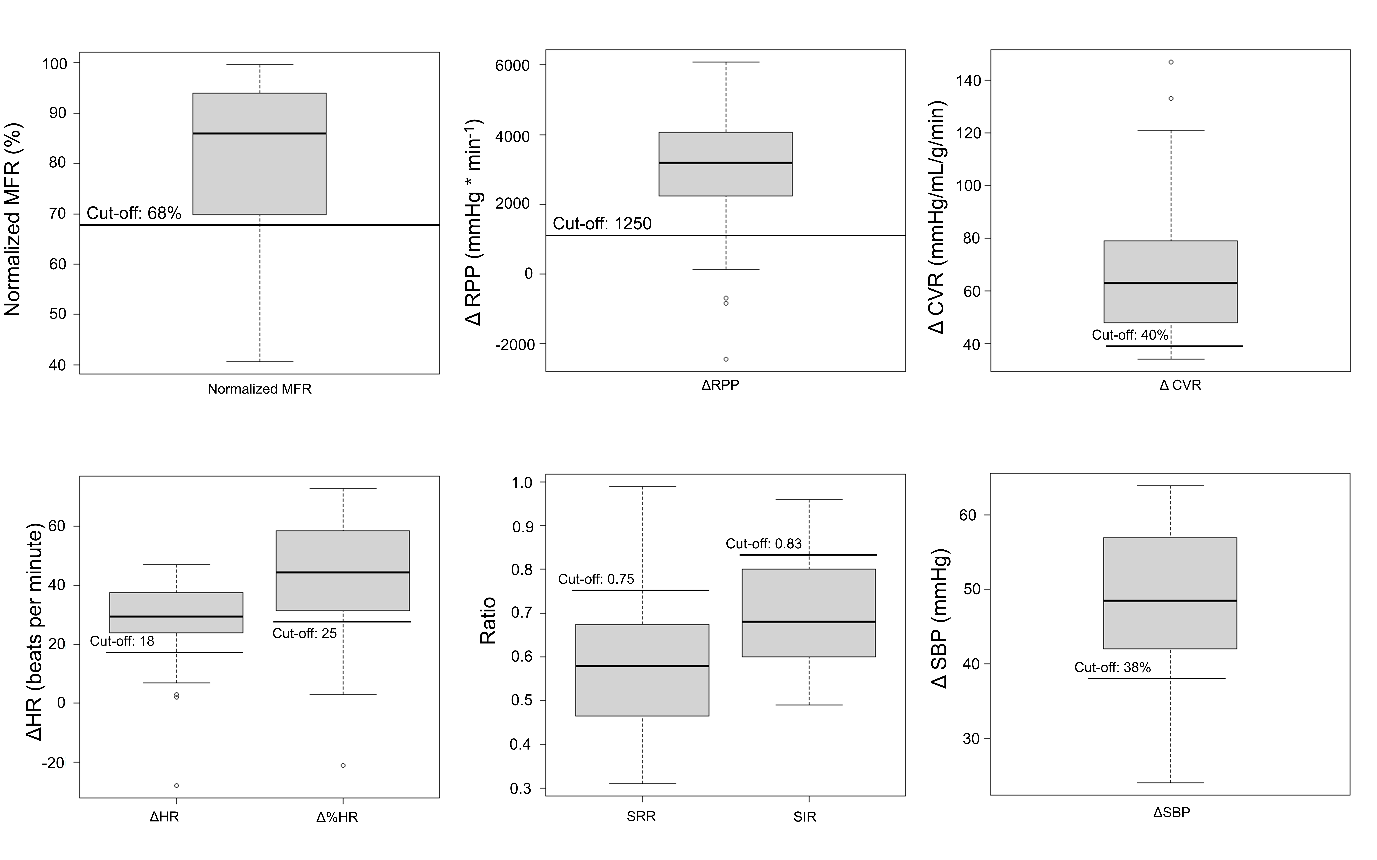
**

MFR = Myocardial Flow reserve, ΔRPP = change in rate pressure product from rest to stress MPI, ΔCVR = the change in the cardiovascular resistance from rest to stress MPI, SRR = Splenic response ratio, SIR = splenic stress-to-rest intensity ratio, ΔHR and Δ%HR = the change in measured and the percentwise heart rate from rest to stress MPI, respectively. ΔSBP = reductions in the systolic blood pressure from rest to stress MPI

**Supplementary material 2. Bar plot of studies with and without consideration of have sufficient hemodynamic response when employing ΔHR.** Sufficient hemodynamic response was declared for ΔHR≥18. The numbers above bars indicate the % wise number of occurrences for the respective measures. The data was divided into two subgroups, studies with and without significant plasma concentrations of caffeine (PCC≥1.0 mg/l and PCC<1.0 mg/l), respectively.


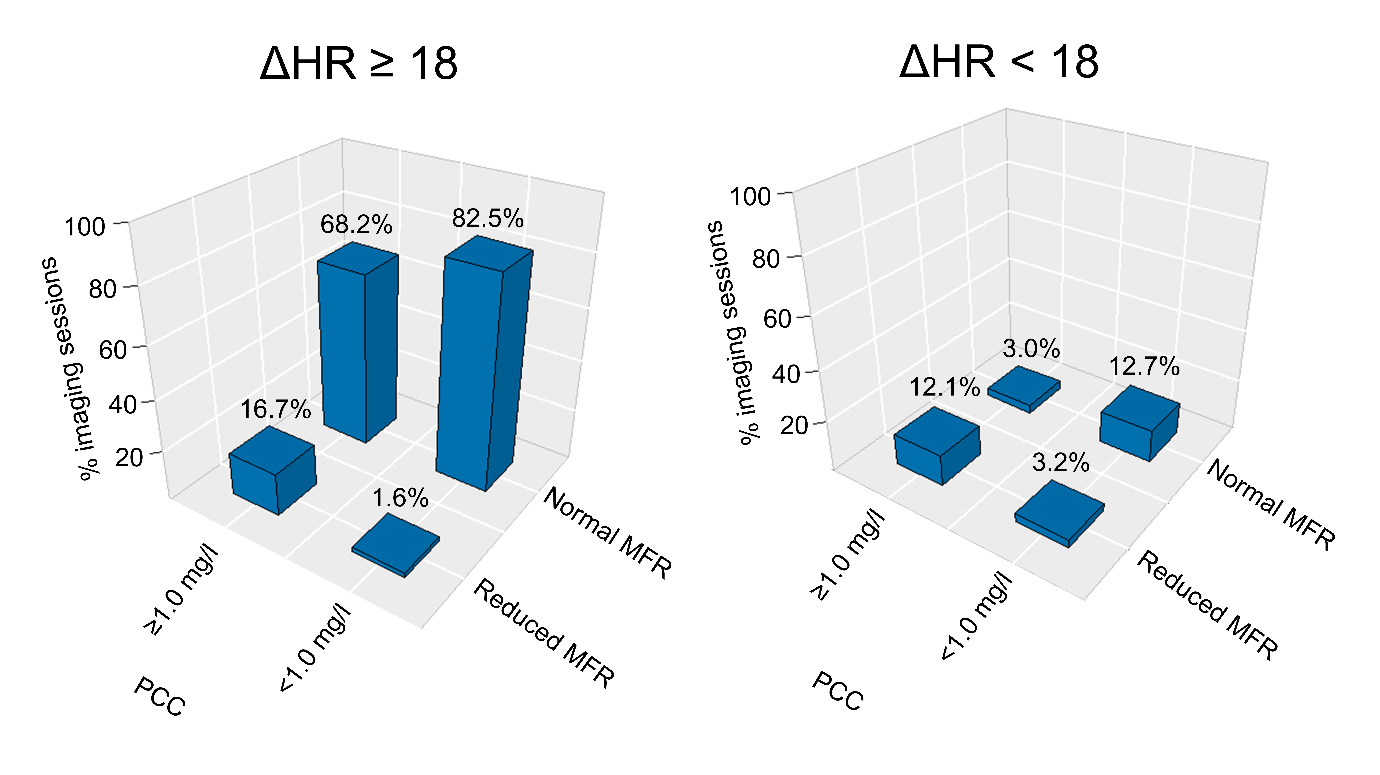


ΔHR = change in heart rate from rest to stress MPI, using HR obtained from ECG-trigger signals, MFR = myocardial flow reserve, PCC = plasma caffeine concentration, MPI = myocardial perfusion imaging

**Supplementary material 3. Bar plot of studies with and without consideration of have sufficient hemodynamic response when employing ΔRPP.** Sufficient hemodynamic response was declared for ΔRPP ≥1250. The numbers above bars indicate the % wise number of occurrences for the respective measures. The data was divided into two subgroups, studies with and without significant plasma concentrations of caffeine (PCC≥1.0 mg/l and PCC<1.0 mg/l), respectively.


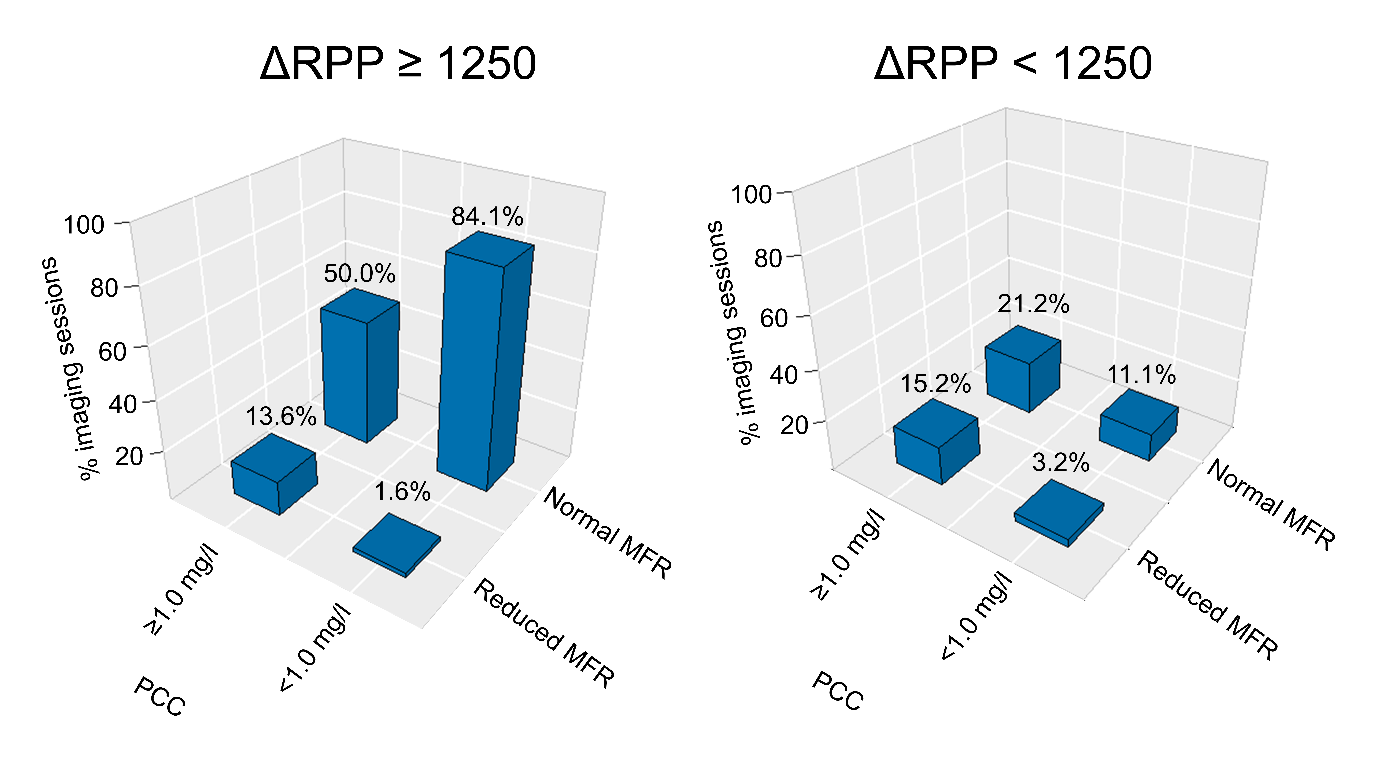


ΔRPP = change in rate pressure product from rest to stress MPI, MFR = myocardial flow reserve, PCC = plasma caffeine concentration, MPI = myocardial perfusion imaging

**Supplementary material 4. Bar plot of studies with and without consideration of have sufficient hemodynamic response when employing ΔSBP.** Sufficient hemodynamic response was declared for ΔSBP≥38. The numbers above bars indicate the % wise number of occurrences for the respective measures. The data was divided into two subgroups, studies with and without significant plasma concentrations of caffeine (PCC≥1.0 mg/l and PCC<1.0 mg/l), respectively.


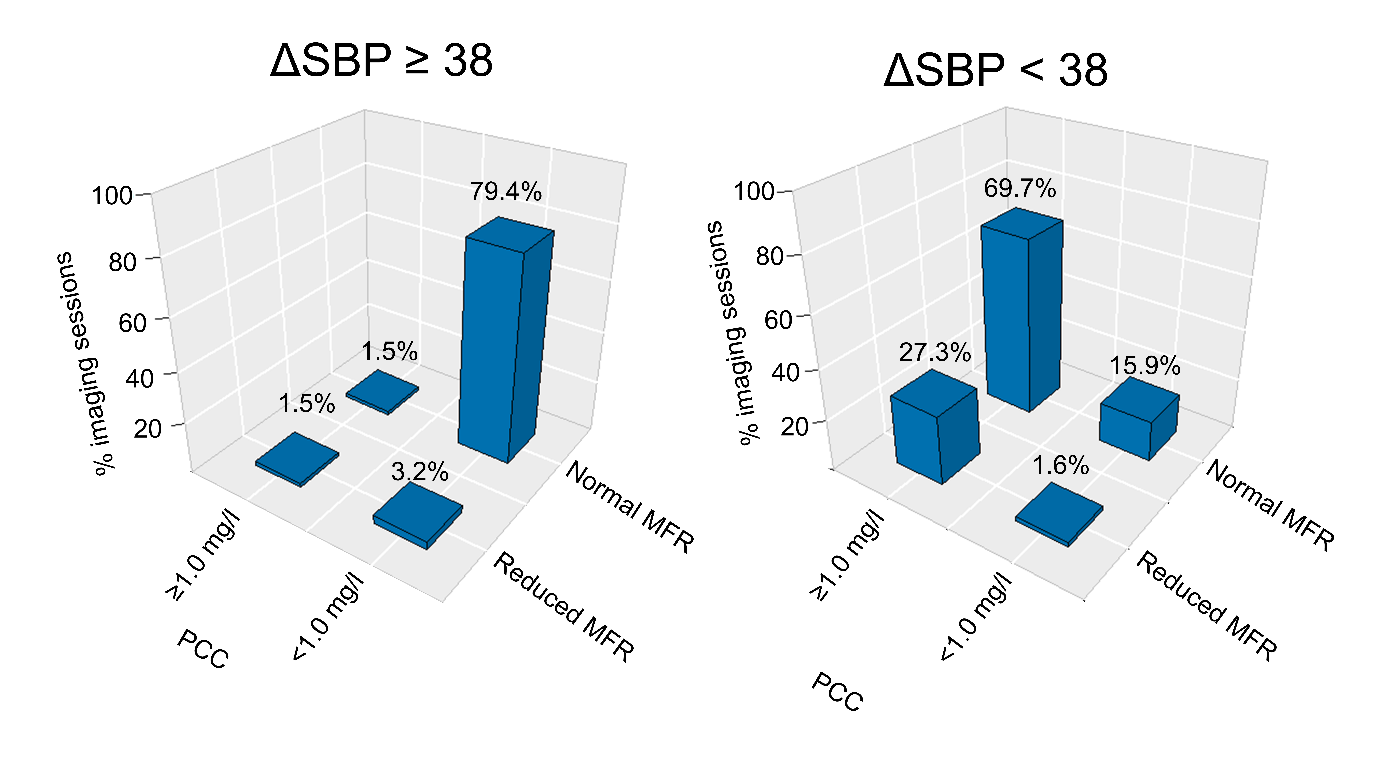


ΔSBP = reductions in the systolic blood pressure from rest to stress MPI, MFR = myocardial flow reserve, PCC = plasma caffeine concentration, MPI = myocardial perfusion imaging.

**Supplementary material 5. Bar plot of studies with and without consideration of have sufficient hemodynamic response when employing ΔCVR.** Sufficient hemodynamic response was declared for ΔCVR≥40%. The numbers above bars indicate the % wise number of occurrences for the respective measures. The data was divided into two subgroups, studies with and without significant plasma concentrations of caffeine (PCC≥1.0 mg/l and PCC<1.0 mg/l), respectively.


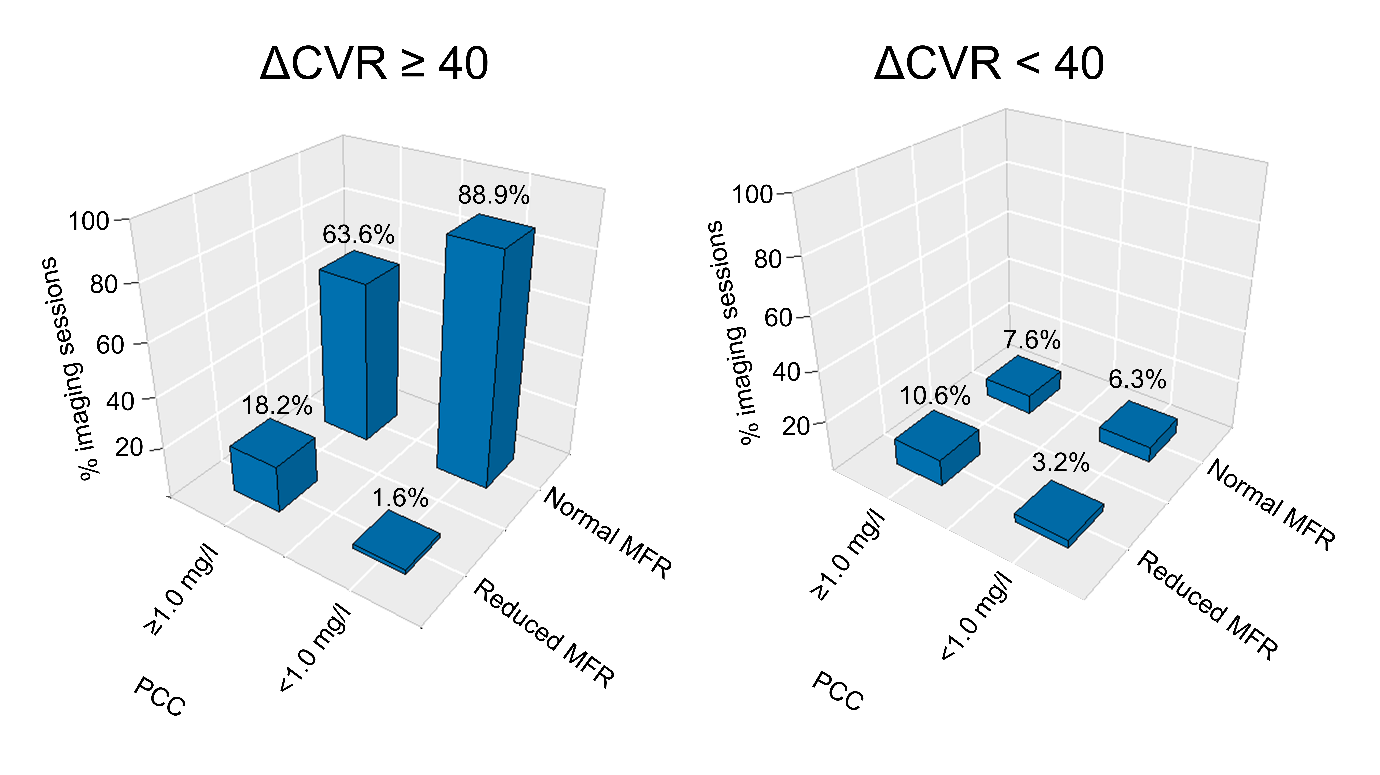


ΔCVR = change in the cardiovascular resistance from rest to stress MPI, MFR = myocardial flow reserve, PCC = plasma caffeine concentration, MPI = myocardial perfusion imaging.
