# Supplementary material for: Image-derived and physiological markers to predict adequate adenosine-induced hyperemic response in Rubidium-82 myocardial perfusion imaging
Source: J Nucl Cardiol. 2022 Feb 11;29(6):3207–17. doi: 10.1007/s12350-022-02906-9 (PMC9834126; doi:10.1007/s12350-022-02906-9)
Supplement: Supplementary file 2 — Supplementary file2 (PPTX 563 kb) [file 12350_2022_2906_MOESM2_ESM.pptx]

## Slide 1
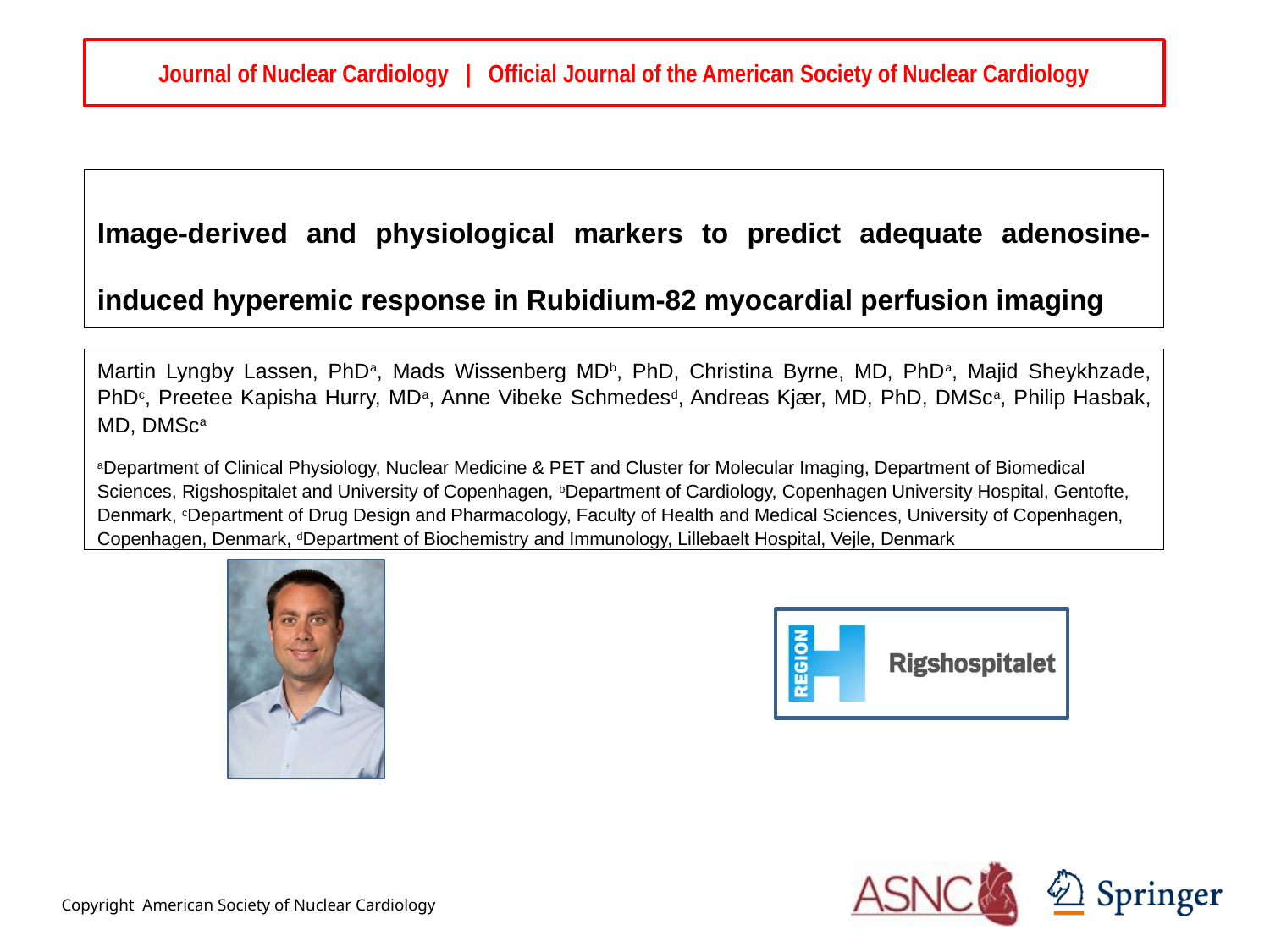

Journal of Nuclear Cardiology | Official Journal of the American Society of Nuclear Cardiology
# Image-derived and physiological markers to predict adequate adenosine-induced hyperemic response in Rubidium-82 myocardial perfusion imaging
Martin Lyngby Lassen, PhDa, Mads Wissenberg MDb, PhD, Christina Byrne, MD, PhDa, Majid Sheykhzade, PhDc, Preetee Kapisha Hurry, MDa, Anne Vibeke Schmedesd, Andreas Kjær, MD, PhD, DMSca, Philip Hasbak, MD, DMSca
aDepartment of Clinical Physiology, Nuclear Medicine & PET and Cluster for Molecular Imaging, Department of Biomedical Sciences, Rigshospitalet and University of Copenhagen, bDepartment of Cardiology, Copenhagen University Hospital, Gentofte, Denmark, cDepartment of Drug Design and Pharmacology, Faculty of Health and Medical Sciences, University of Copenhagen, Copenhagen, Denmark, dDepartment of Biochemistry and Immunology, Lillebaelt Hospital, Vejle, Denmark
Copyright American Society of Nuclear Cardiology

## Slide 2
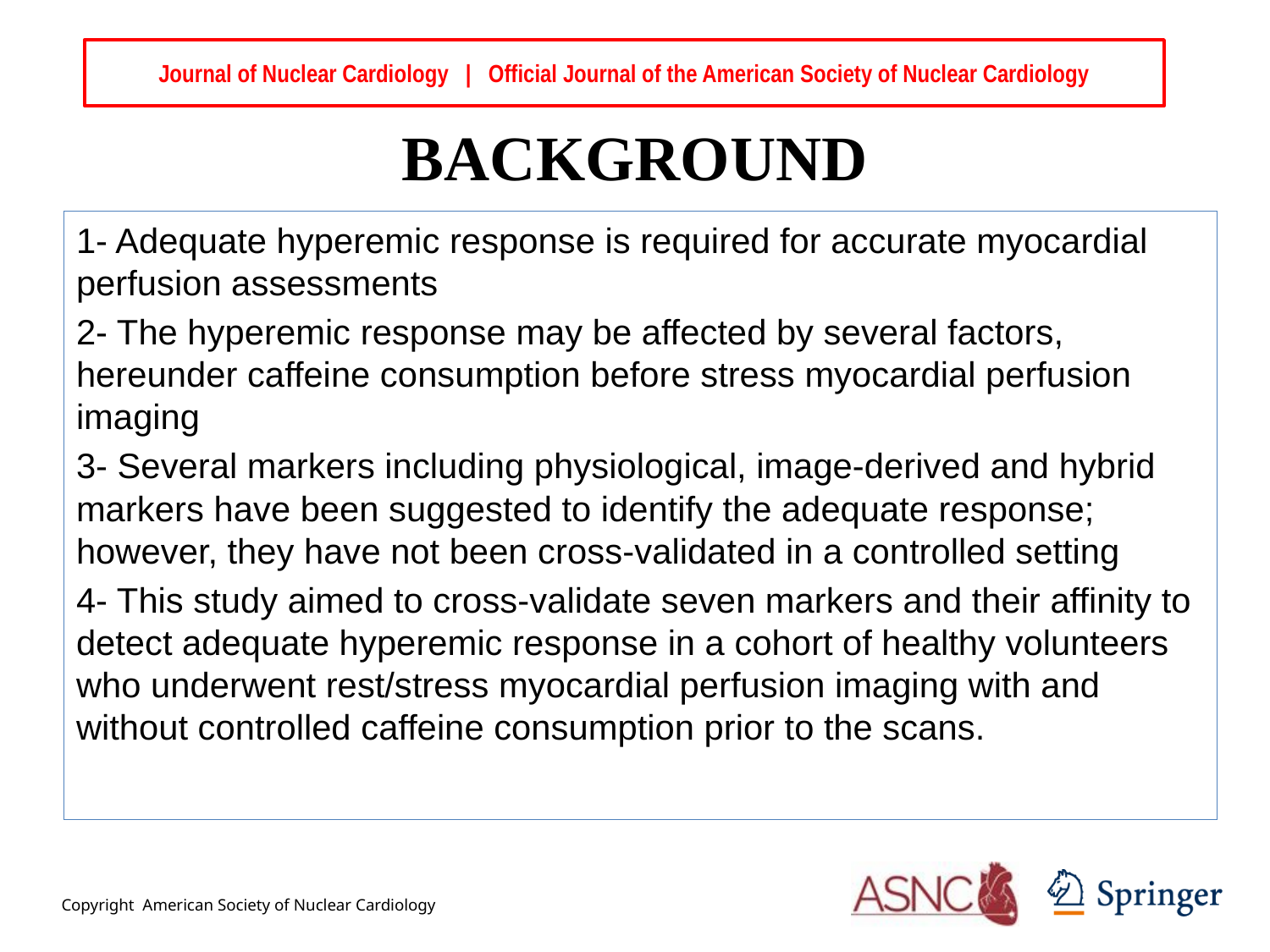

Journal of Nuclear Cardiology | Official Journal of the American Society of Nuclear Cardiology
# BACKGROUND
1- Adequate hyperemic response is required for accurate myocardial perfusion assessments
2- The hyperemic response may be affected by several factors, hereunder caffeine consumption before stress myocardial perfusion imaging
3- Several markers including physiological, image-derived and hybrid markers have been suggested to identify the adequate response; however, they have not been cross-validated in a controlled setting
4- This study aimed to cross-validate seven markers and their affinity to detect adequate hyperemic response in a cohort of healthy volunteers who underwent rest/stress myocardial perfusion imaging with and without controlled caffeine consumption prior to the scans.
Copyright American Society of Nuclear Cardiology

## Slide 3
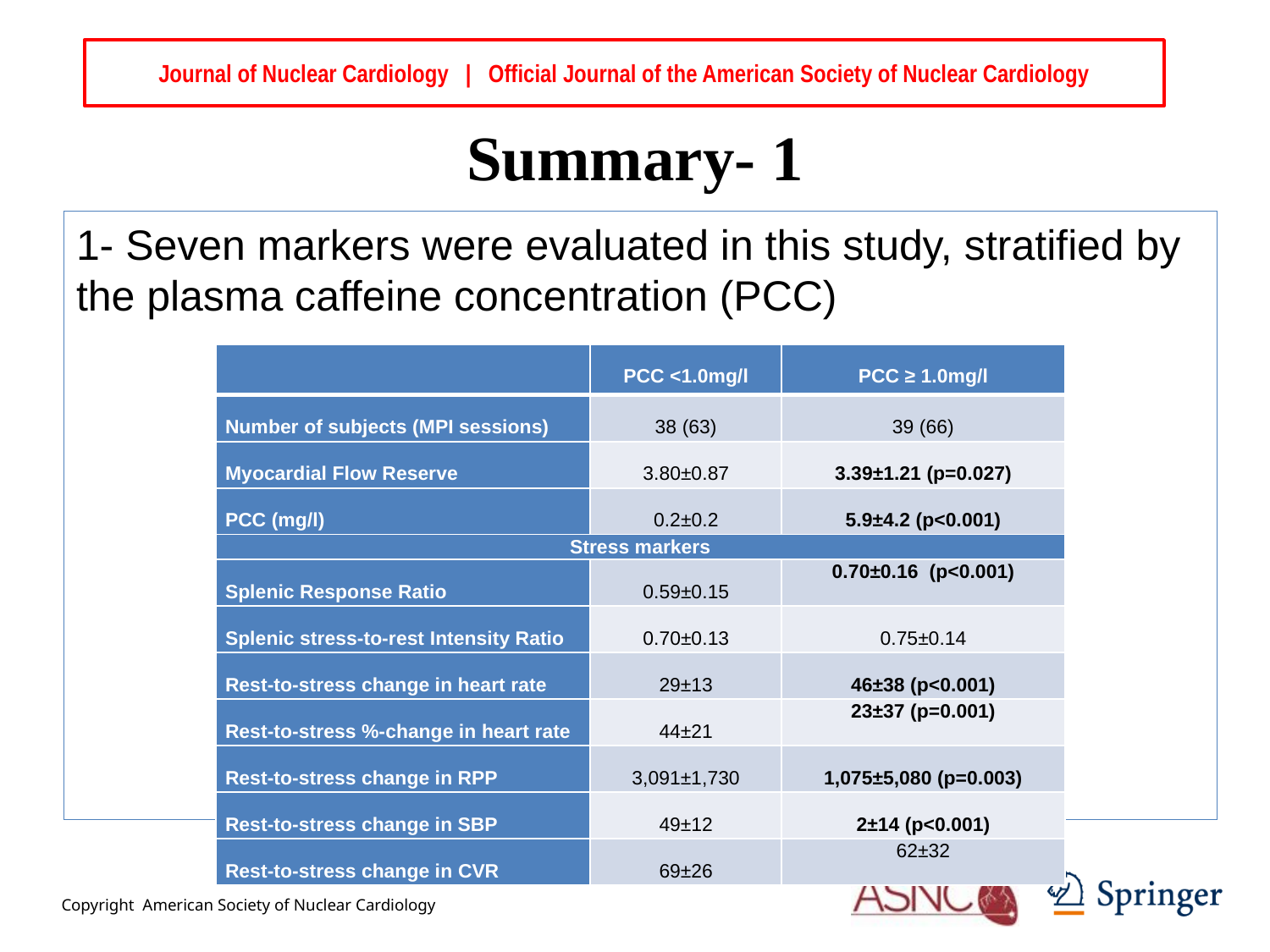

Journal of Nuclear Cardiology | Official Journal of the American Society of Nuclear Cardiology
# Summary- 1
1- Seven markers were evaluated in this study, stratified by the plasma caffeine concentration (PCC)
| | PCC <1.0mg/l | PCC ≥ 1.0mg/l |
| --- | --- | --- |
| Number of subjects (MPI sessions) | 38 (63) | 39 (66) |
| Myocardial Flow Reserve | 3.80±0.87 | 3.39±1.21 (p=0.027) |
| PCC (mg/l) | 0.2±0.2 | 5.9±4.2 (p<0.001) |
| Stress markers | | |
| Splenic Response Ratio | 0.59±0.15 | 0.70±0.16 (p<0.001) |
| Splenic stress-to-rest Intensity Ratio | 0.70±0.13 | 0.75±0.14 |
| Rest-to-stress change in heart rate | 29±13 | 46±38 (p<0.001) |
| Rest-to-stress %-change in heart rate | 44±21 | 23±37 (p=0.001) |
| Rest-to-stress change in RPP | 3,091±1,730 | 1,075±5,080 (p=0.003) |
| Rest-to-stress change in SBP | 49±12 | 2±14 (p<0.001) |
| Rest-to-stress change in CVR | 69±26 | 62±32 |
Copyright American Society of Nuclear Cardiology

## Slide 4
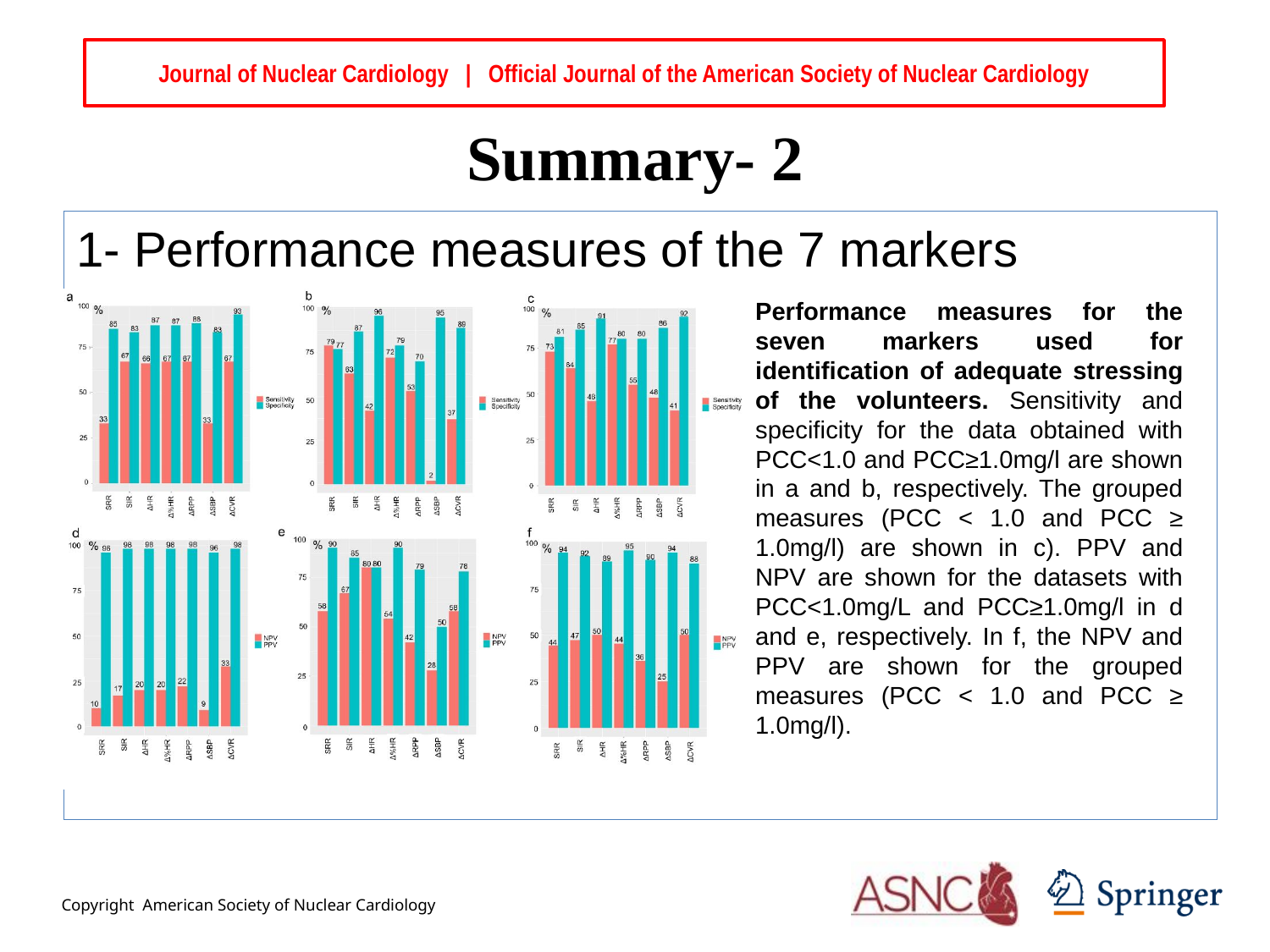

Journal of Nuclear Cardiology | Official Journal of the American Society of Nuclear Cardiology
# Summary- 2
1- Performance measures of the 7 markers
Performance measures for the seven markers used for identification of adequate stressing of the volunteers. Sensitivity and specificity for the data obtained with PCC<1.0 and PCC≥1.0mg/l are shown in a and b, respectively. The grouped measures (PCC < 1.0 and PCC ≥ 1.0mg/l) are shown in c). PPV and NPV are shown for the datasets with PCC<1.0mg/L and PCC≥1.0mg/l in d and e, respectively. In f, the NPV and PPV are shown for the grouped measures (PCC < 1.0 and PCC ≥ 1.0mg/l).
Copyright American Society of Nuclear Cardiology

## Slide 5
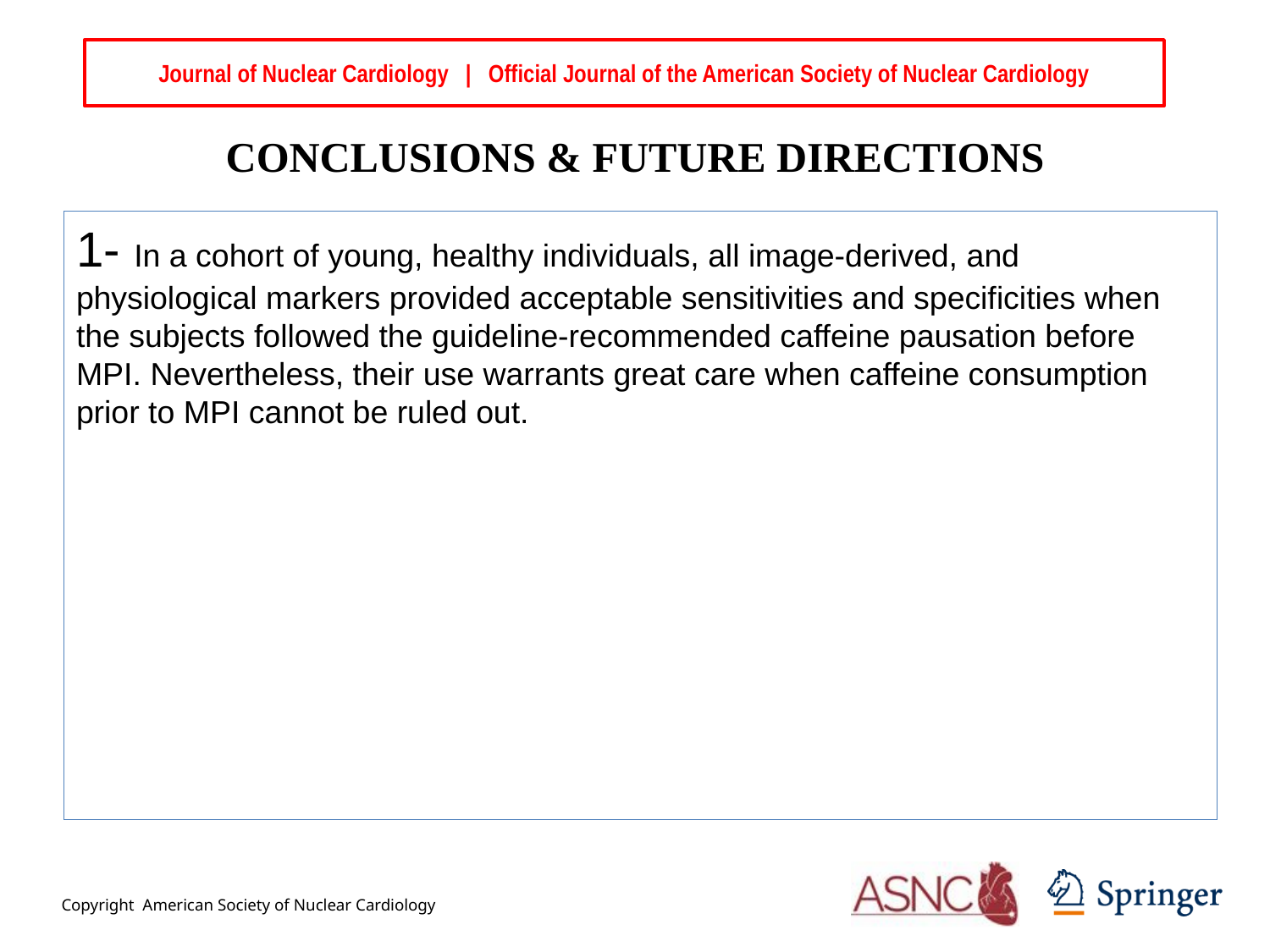

Journal of Nuclear Cardiology | Official Journal of the American Society of Nuclear Cardiology
# CONCLUSIONS & FUTURE DIRECTIONS
1- In a cohort of young, healthy individuals, all image-derived, and physiological markers provided acceptable sensitivities and specificities when the subjects followed the guideline-recommended caffeine pausation before MPI. Nevertheless, their use warrants great care when caffeine consumption prior to MPI cannot be ruled out.
Copyright American Society of Nuclear Cardiology
