# Supplementary figures and images for: Image-derived and physiological markers to predict adequate adenosine-induced hyperemic response in Rubidium-82 myocardial perfusion imaging
Source: J Nucl Cardiol. 2022 Feb 11;29(6):3207–17. doi: 10.1007/s12350-022-02906-9 (PMC9834126; doi:10.1007/s12350-022-02906-9)

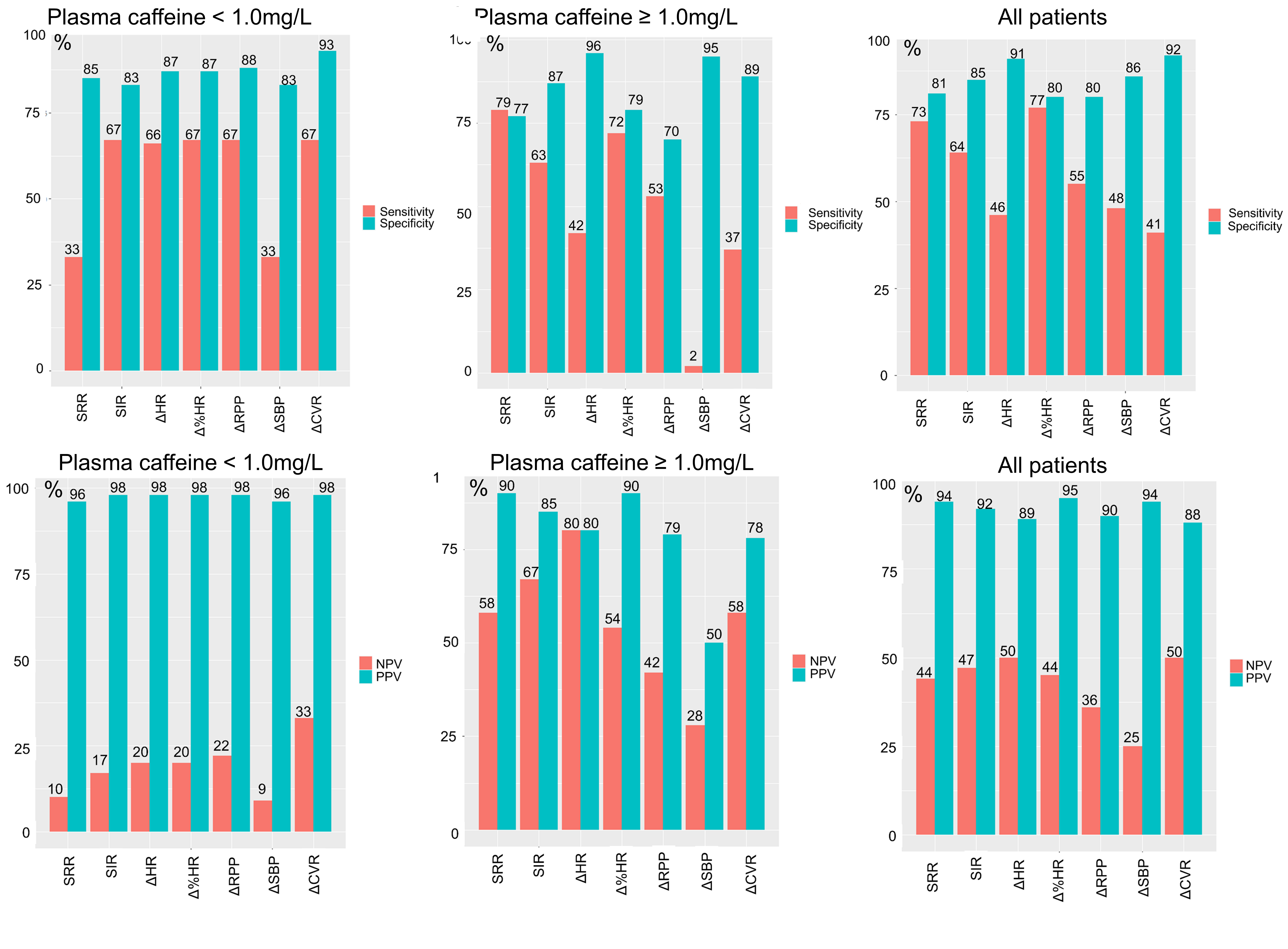

Supplement: Supplementary file 3 — Supplementary file3 (TIF 8083 kb) [file 12350_2022_2906_MOESM3_ESM.tif]
